# Supplementary material for: Multi‐dataset identification of innovative feature genes and molecular mechanisms in keratoconus
Source: J Cell Mol Med. 2024 Sep 19;28(18):e70079. doi: 10.1111/jcmm.70079 (PMC11412914; doi:10.1111/jcmm.70079)
Supplement: Supplementary file 2 — Material S2. [file JCMM-28-e70079-s002.zip › 7.GSEA analysis/1.ARL11/KEGG_EPITHELIAL_CELL_SIGNALING_IN_HELICOBACTER_PYLORI_INFECTION.html]

Details for gene set KEGG\_EPITHELIAL\_CELL\_SIGNALING\_IN\_HELICOBACTER\_PYLORI\_INFECTION[GSEA]

|  || Dataset | ARL11.group.cls#h\_versus\_l |
| Phenotype | group.cls#h\_versus\_l |
| Upregulated in class | l |
| GeneSet | KEGG\_EPITHELIAL\_CELL\_SIGNALING\_IN\_HELICOBACTER\_PYLORI\_INFECTION |
| Enrichment Score (ES) | -0.26545548 |
| Normalized Enrichment Score (NES) | -0.92420083 |
| Nominal p-value | 0.5390625 |
| FDR q-value | 1.0 |
| FWER p-Value | 1.0 |
Table: GSEA Results Summary

  

Fig 1: Enrichment plot: KEGG\_EPITHELIAL\_CELL\_SIGNALING\_IN\_HELICOBACTER\_PYLORI\_INFECTION      
 Profile of the Running ES Score & Positions of GeneSet Members on the Rank Ordered List

  

| SYMBOL | TITLE | RANK IN GENE LIST | RANK METRIC SCORE | RUNNING ES | CORE ENRICHMENT || 1 | CXCR2 | na | 1957 | 0.299 | 0.0033 | No |
| 2 | ATP6V0A4 | na | 4084 | 0.198 | -0.0185 | No |
| 3 | PLCG1 | na | 4334 | 0.189 | 0.0120 | No |
| 4 | CCL5 | na | 4596 | 0.179 | 0.0402 | No |
| 5 | PLCG2 | na | 6096 | 0.135 | 0.0239 | No |
| 6 | ATP6V0D1 | na | 6786 | 0.117 | 0.0273 | No |
| 7 | IKBKB | na | 8698 | 0.079 | -0.0120 | No |
| 8 | CASP3 | na | 9607 | 0.066 | -0.0251 | No |
| 9 | EGFR | na | 11115 | 0.046 | -0.0594 | No |
| 10 | MAPK9 | na | 11167 | 0.046 | -0.0517 | No |
| 11 | MAPK11 | na | 11276 | 0.044 | -0.0460 | No |
| 12 | CSK | na | 11826 | 0.038 | -0.0543 | No |
| 13 | CXCL1 | na | 12091 | 0.035 | -0.0548 | No |
| 14 | ATP6V1B2 | na | 12446 | 0.032 | -0.0587 | No |
| 15 | ATP6AP1 | na | 12793 | 0.029 | -0.0629 | No |
| 16 | MAPK12 | na | 13078 | 0.026 | -0.0658 | No |
| 17 | ATP6V1E2 | na | 13257 | 0.025 | -0.0660 | No |
| 18 | JUN | na | 13319 | 0.024 | -0.0629 | No |
| 19 | ATP6V1B1 | na | 14360 | 0.016 | -0.0897 | No |
| 20 | GIT1 | na | 14382 | 0.016 | -0.0871 | No |
| 21 | ATP6V0E2 | na | 14410 | 0.016 | -0.0848 | No |
| 22 | ATP6V1G3 | na | 14530 | 0.015 | -0.0853 | No |
| 23 | PTPN11 | na | 14607 | 0.014 | -0.0846 | No |
| 24 | ATP6V0A1 | na | 15062 | 0.011 | -0.0956 | No |
| 25 | CHUK | na | 15127 | 0.010 | -0.0954 | No |
| 26 | MAPK10 | na | 15299 | 0.009 | -0.0985 | No |
| 27 | ATP6V0B | na | 15548 | 0.007 | -0.1042 | No |
| 28 | TJP1 | na | 15790 | 0.005 | -0.1102 | No |
| 29 | RAC1 | na | 16390 | 0.001 | -0.1272 | No |
| 30 | ATP6V1F | na | 17512 | -0.007 | -0.1582 | No |
| 31 | MAPK14 | na | 17567 | -0.007 | -0.1583 | No |
| 32 | ATP6V0C | na | 17696 | -0.008 | -0.1604 | No |
| 33 | MAP2K4 | na | 17706 | -0.008 | -0.1590 | No |
| 34 | CDC42 | na | 18361 | -0.013 | -0.1753 | No |
| 35 | F11R | na | 18558 | -0.014 | -0.1782 | No |
| 36 | ATP6V1C1 | na | 18651 | -0.015 | -0.1779 | No |
| 37 | ATP6V0E1 | na | 19061 | -0.017 | -0.1862 | No |
| 38 | PTPRZ1 | na | 19734 | -0.022 | -0.2012 | No |
| 39 | ATP6V1D | na | 20147 | -0.025 | -0.2082 | No |
| 40 | ATP6V1G2 | na | 20600 | -0.028 | -0.2156 | No |
| 41 | ATP6V1A | na | 20723 | -0.029 | -0.2133 | No |
| 42 | MAPK13 | na | 20970 | -0.031 | -0.2142 | No |
| 43 | ATP6V1G1 | na | 21288 | -0.033 | -0.2167 | No |
| 44 | IKBKG | na | 22799 | -0.048 | -0.2507 | No |
| 45 | NOD1 | na | 22856 | -0.049 | -0.2426 | No |
| 46 | PAK1 | na | 23649 | -0.058 | -0.2539 | Yes |
| 47 | ATP6V1E1 | na | 23880 | -0.061 | -0.2485 | Yes |
| 48 | SRC | na | 24048 | -0.063 | -0.2407 | Yes |
| 49 | ATP6V0D2 | na | 24169 | -0.065 | -0.2313 | Yes |
| 50 | NFKBIA | na | 24454 | -0.069 | -0.2258 | Yes |
| 51 | MAPK8 | na | 24525 | -0.070 | -0.2139 | Yes |
| 52 | ADAM17 | na | 24531 | -0.070 | -0.2001 | Yes |
| 53 | ATP6V1C2 | na | 25402 | -0.081 | -0.2089 | Yes |
| 54 | ATP6V1H | na | 25843 | -0.088 | -0.2041 | Yes |
| 55 | LYN | na | 25913 | -0.089 | -0.1883 | Yes |
| 56 | HBEGF | na | 25935 | -0.089 | -0.1711 | Yes |
| 57 | ADAM10 | na | 26044 | -0.091 | -0.1561 | Yes |
| 58 | NFKB1 | na | 26767 | -0.104 | -0.1563 | Yes |
| 59 | MET | na | 27578 | -0.120 | -0.1556 | Yes |
| 60 | MAP3K14 | na | 28361 | -0.140 | -0.1503 | Yes |
| 61 | RELA | na | 29186 | -0.162 | -0.1417 | Yes |
| 62 | CXCL8 | na | 29280 | -0.165 | -0.1115 | Yes |
| 63 | IGSF5 | na | 30323 | -0.199 | -0.1019 | Yes |
| 64 | JAM2 | na | 33406 | -0.352 | -0.1207 | Yes |
| 65 | CXCR1 | na | 33444 | -0.355 | -0.0508 | Yes |
| 66 | JAM3 | na | 34122 | -0.440 | 0.0175 | Yes |
Table: GSEA details [plain text format]

  

Fig 2: KEGG\_EPITHELIAL\_CELL\_SIGNALING\_IN\_HELICOBACTER\_PYLORI\_INFECTION      
 Blue-Pink O' Gram in the Space of the Analyzed GeneSet

  

Fig 3: KEGG\_EPITHELIAL\_CELL\_SIGNALING\_IN\_HELICOBACTER\_PYLORI\_INFECTION: Random ES distribution      
 Gene set null distribution of ES for **KEGG\_EPITHELIAL\_CELL\_SIGNALING\_IN\_HELICOBACTER\_PYLORI\_INFECTION**
